# *Sparassis crispa* Intake Improves the Reduced Lipopolysaccharide-Induced TNF-α Production That Occurs upon Exhaustive Exercise in Mice

**DOI:** 10.3390/nu11092049

**Published:** 2019-09-02

**Authors:** Masataka Uchida, Naoki Horii, Natsuki Hasegawa, Eri Oyanagi, Hiromi Yano, Motoyuki Iemitsu

**Affiliations:** 1 Faculty of Sport and Health Science, Ritsumeikan University, 1-1-1 Nojihigashi, Kusatsu, Shiga 525-8577, Japan; 2 Research Fellow of Japan Society for the Promotion of Science, 5-3-1 Kojimachi, Chiyoda-ku, Tokyo 102-0083, Japan; 3 Research Organization of Science and Technology, Ritsumeikan University, 1-1-1 Nojihigashi, Kusatsu, Shiga 525-8577, Japan; 4 Department of Health and Sports Science, Kawasaki University of Medical Welfare, Kurashiki, Okayama 701-0193, Japan

**Keywords:** exhaustive exercise, immunodepression, small intestine, toll-like receptor 4

## Abstract

Our previous study showed that lipopolysaccharide (LPS)-induced tumor necrosis factor (TNF)-α production is inhibited by acute exhaustive exercise in mice, leading to transient immunodepression after exercise. *Sparassis crispa* (SC), an edible mushroom, has immunopotentiative properties. This study aimed to clarify the effects of SC intake on reduced LPS-induced TNF-α production upon exhaustive exercise in mice. Male C3H/HeN mice were randomly divided into three groups: normal chow intake + resting sedentary, normal chow intake + acute exhaustive treadmill running exercise, and SC intake (chow containing 5% SC powder for 8 weeks) + the exhaustive exercise groups. Each group was injected with LPS immediately after the exhaustive exercise or rest. Plasma and tissue TNF-α levels were significantly decreased by exhaustive exercise. However, this reduction of the TNF-α level was partially attenuated in the plasma and small intestine by SC intake. Although levels of TLR4 and MyD88 protein expression were significantly decreased in tissues by exhaustive exercise, the reduction of TLR4 and MyD88 levels in the small intestine was partially attenuated by SC intake. These results suggest that SC intake attenuates exhaustive exercise-induced reduction of TNF-α production via the retention of TLR4 and MyD88 expression in the small intestine.

## 1. Introduction

Strenuous exercise transiently reduces immune function, resulting in an increased risk of infection, such as upper respiratory tract infections, in athletes [1,2]. Since exhaustive exercise inhibits systemic tumor necrosis factor-α (TNF-α) production that normally occurs in a bacterial infection model (injection of lipopolysaccharide [LPS]), immunodepression after strenuous exercise is involved in increased susceptibility to microorganism infection [1,2,3]. Therefore, preventing exhaustive exercise-induced immunodepression is important for athletes and exercise enthusiasts.

Pharmacological and pathogenic immunodepression is improved in mice by supplementation with β-glucan and vitamin D extracted from mushrooms [4,5]. *Sparassis crispa* is an edible mushroom that contains high amounts of β-glucan, more than 30% of the dry weight of the fruiting bodies, compared with levels in other mushrooms [6]. Several studies have revealed that consumption of *S. crispa* and β-glucan extracted from *S. crispa* has immunopotentiative effects, such as enhanced inflammatory cytokine production in splenocytes, improved peripheral NK cell activity, and anti-tumor action [6,7,8,9]. Based on these results, *S. crispa* consumption enhances immune function. Therefore, we hypothesized that *S. crispa* consumption may improve acute exhaustive exercise-induced immunodepression. However, the effect of *S. crispa* intake on exhaustive exercise-induced reduction of immune response to bacterial infection is unclear.

Therefore, the aim of this study was to investigate the effects of *S. crispa* intake on reduced LPS-induced TNF-α production that occurs upon exhaustive exercise, both systemically and in various tissues of mice. Moreover, to investigate the molecular mechanism of the effect of *S. crispa*, we examined LPS-induced activation of the Toll-like receptor 4 (TLR4) signal pathway in each tissue.

## 2. Materials and Methods

### 2.1. Animals and Protocol

Four-week-old male C3H/HeN mice were obtained from Clea Japan (Tokyo, Japan). The ethical approval for this study was obtained from the Committee on Animal Care at Ritsumeikan University in Japan (BKC2017-015). All mice were housed in a controlled environment (20 ± 1 °C, 12:12 h light: dark cycle). Mice were randomly divided into 3 groups: Normal diet + sedentary (CD + Sed, *n* = 8) group, normal diet + exercise (CD + Ex, *n* = 10) group, and *S. crispa* diet + exercise (SD + Ex, *n* = 10) group. The CD + Sed and CD + Ex groups were given access to water and fed a normal diet (CE-2; CLEA Japan, Tokyo, Japan) ad libitum during the 8-week diet intervention period. The SD + Ex group was fed CE-2 containing 5% *S. crispa* fruiting body dry powder (Kyoei-Seimitsu, Shiga, Japan) during the same diet intervention period. 

After diet intervention, the CD + Ex and SD + Ex groups were run on a treadmill to exhaustion, and the normal diet + non-exercise group was maintained in a sedentary condition. Immediately after the exercise or sedentary period, the mice were lightly anesthetized with isoflurane and injected with 1 mg/kg LPS (100 μL/mouse) in the orbital vein. Blood samples were obtained from the abdominal vein under general anesthesia with 2% isoflurane 1 h after LPS injection. After sacrifice, the lung, liver, spleen, small intestine and large intestine were collected, frozen in liquid nitrogen, and stored at −80 °C until further analysis. Gastrocnemius muscle mass, soleus muscle mass, plantaris muscle mass, tibialis anterior muscle mass and extensor digitorum longus muscle mass were measured.

### 2.2. Drugs

LPS (*Escherichia coli* 055:B5) was obtained from Sigma (St. Louis, MO, USA), dissolved in pyrogen-free 0.9% NaCl at a concentration of 1 mg/mL, and kept frozen at −20 °C as a stock solution.

### 2.3. Exercise Protocol

The exercise protocol was as follows [10,11]: Mice ran on a treadmill at a starting speed of 9 m/minute. The speed was increased by 2 m/minute every three minutes until 17 m/minute. Thereafter, we continued to increase the speed by 1 m/minute every three minutes until exhaustion. Exhaustion was defined as the point at which a mouse refused to run despite being lightly touched. No electric shocks were used during the treadmill runs. In this experiment, the mean running times in the CD + Ex and SD + Ex groups were 69 ± 7 and 72 ± 4 min, respectively (N.S.). The CD + N group was maintained in a sedentary condition for 60–70 min without access to food and water.

### 2.4. Enzyme-Linked Immunosorbent Assay for TNF-α

Plasma and tissue TNF-α concentrations were measured by an enzyme-linked immunosorbent assay (ELISA) using a commercially available murine kit (R&D Systems, Minneapolis, MN, USA). The absorbance was measured at 450 nm by microplate reader using an xMark microplate spectrophotometer (Bio-Rad Laboratories, Hercules, CA, USA) and was proportional to the concentration of TNF-α in the sample by using linear fit of the log–log plot of the standard curve.

### 2.5. Immunoblot Analysis

Western blot analysis was performed to assess TLR4 and MyD88 protein expression levels as previously described [12]. Briefly, protein samples from the lung, liver, spleen, small intestine, and large intestine were separated by 10% SDS-polyacrylamide gel and transferred to polyvinylidene difluoride (PVDF) membranes (Millipore, Billerica, MA, USA). The membranes were treated for 1 h with blocking buffer (5% skim milk in phosphate-buffered saline with 0.1% Tween 20 [PBS-T]) and then incubated for 12 h in blocking buffer at 4 °C with antibodies (diluted 1:1000 in blocking buffer) against TLR4 (#14358, Cell Signaling Technology, Danvers, MA, USA), MyD88 (#4283, Cell Signaling Technology) or β-actin (#4967, Cell Signaling Technology). The membranes were washed three times with PBS-T, and then incubated for 1 h at room temperature (22–24 °C) with horseradish peroxidase (HRP)-conjugated anti-rabbit secondary antibody (#7074, Cell Signaling Technology) diluted 1:3000 in blocking buffer. The membranes were then washed three times with PBS-T for 30 min. Finally, the Myeloid differentiation primary response 88 (MyD88) level was detected using the Immobilon Forte Western HRP substrate (Millipore), and the TLR4 level was detected using the Enhanced Chemiluminescence Plus system (GE Healthcare Biosciences, AB, USA). TLR4 and MyD88 levels were visualized on a FUSION-Chemiluminescence Imaging System (Vilber-Lourmat, Marne, Cedex, France). Densitometry was performed using ImageJ software (ver 1.48; National Institutes of Health, Bethesda, MD, USA).

### 2.6. Statistical Analysis

All values are expressed as the mean ± SEM. Statistical evaluations were performed using one-way ANOVA. Fisher’s post-hoc test was used to correct for multiple comparisons when analyses revealed significant differences. Data were considered to be significantly different when *p*-values were less than 0.05.

## 3. Results

### 3.1. Animal Study

#### 3.1.1. Animal Characteristics

No significant differences in body weight or food intake were found among CD + Sed, CD + Ex and SD + Ex groups (Table 1). Additionally, no significant differences in heart mass, gastrocnemius muscle mass, soleus muscle mass, plantaris muscle mass, tibialis anterior muscle mass and extensor digitorum longus muscle mass were observed among the CD + Sed, CD + Ex and SD + Ex groups (Table 1).

#### 3.1.2. Plasma TNF-α Concentration in Response to LPS Stimulation

**Figure 1:**
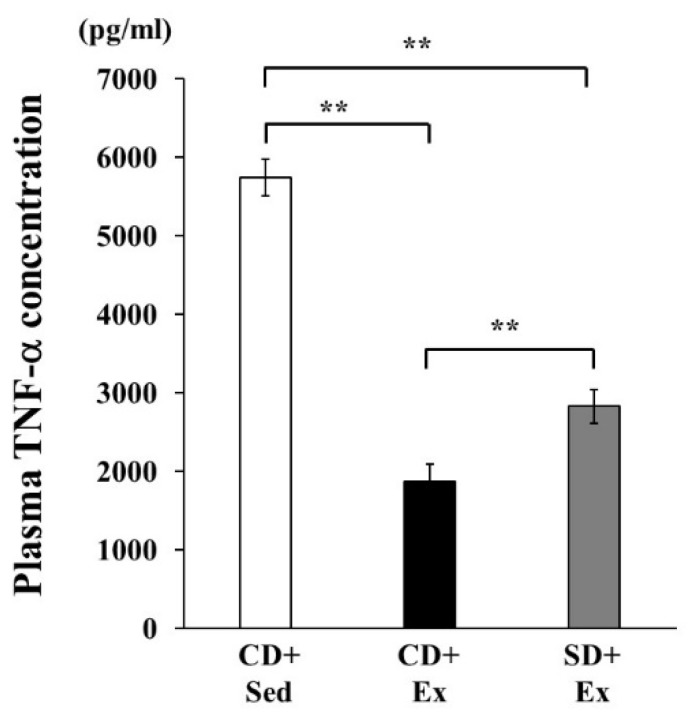
The effect of exhaustive exercise and *Sparassis crispa* intake on plasma tumor necrosis factor (TNF)-α concentration in response to lipopolysaccharide (LPS) injection in mice. Values are means ± SEM. CD + Sed (*n* = 8): normal diet + sedentary, CD + Ex (*n* = 10): normal diet + exercise, SD + Ex (*n* = 10): *S. crispa* diet + exercise. ** *p* < 0.01.

The plasma TNF-α concentration in Ex groups was significantly lower than that in the CD + Sed group at 1 h after LPS injection, as seen in Figure 1. Plasma TNF-α concentration in the SD + Ex group was significantly higher than that in the CD + Ex group at 1 h after LPS injection, as seen in Figure 1. 

#### 3.1.3. Effect of *S. crispa* Intake and Exhaustive Exercise on TNF-α Concentration in Mouse Tissues in Response to LPS

**Figure 2:**
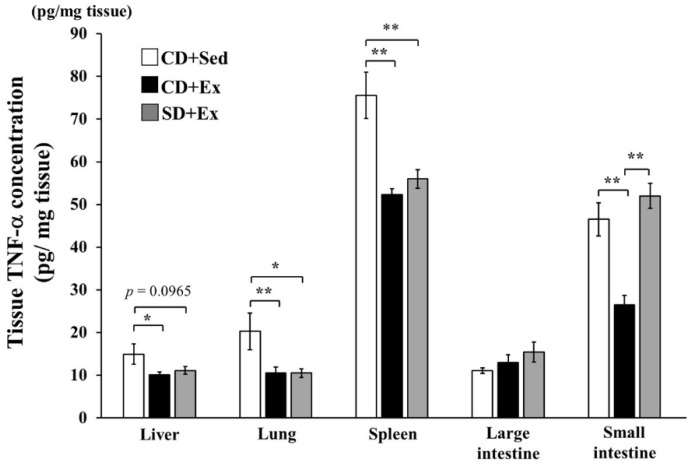
The effect of exhaustive exercise and *Sparassis crispa* intake on TNF-α concentration in the liver, lung, spleen, large intestine and small intestine of mice. Values are means ± SEM. * *p* < 0.05, ** *p* < 0.01.

TNF-α concentration in the CD + Ex and SD + Ex groups was significantly lower in the liver, lung and spleen tissues than that in the CD + Sed group at 1 after LPS injection (Figure 2), while no significant differences between CD + Ex and SD + Ex were observed (Figure 2). In the small intestine, TNF-α concentration was significantly decreased in the CD + Ex and SD + Ex groups as compared with that in the CD + Sed group (Figure 2). However, TNF-α concentration in the small intestine of mice in the SD + Ex group was higher than that in the CD + Ex group (Figure 2). No significant difference in TNF-α concentration was observed across the three groups in the large intestine.

#### 3.1.4. Effect of *S. crispa* Intake and Exhaustive Exercise on TLR4 Signaling in Mouse Tissues in Response to LPS

LPS stimulation induces TNF-α production by the TLR4 via activation of MyD88. We sought to clarify the effect of *S. crispa* intake on TLR4 and its downstream signaling after exhaustive exercise. TLR4 protein levels in the liver, lung and small intestine were significantly lower in the CD + Ex group compared to that in the CD + Sed group. The TLR4 protein level was lower in the liver and lung tissues of the SD + Ex group compared to that in the CD + Sed group, whereas TLR4 protein levels in the small intestine were higher in the SD + Ex group compared to those in the CD + Ex group.

**Figure 3:**
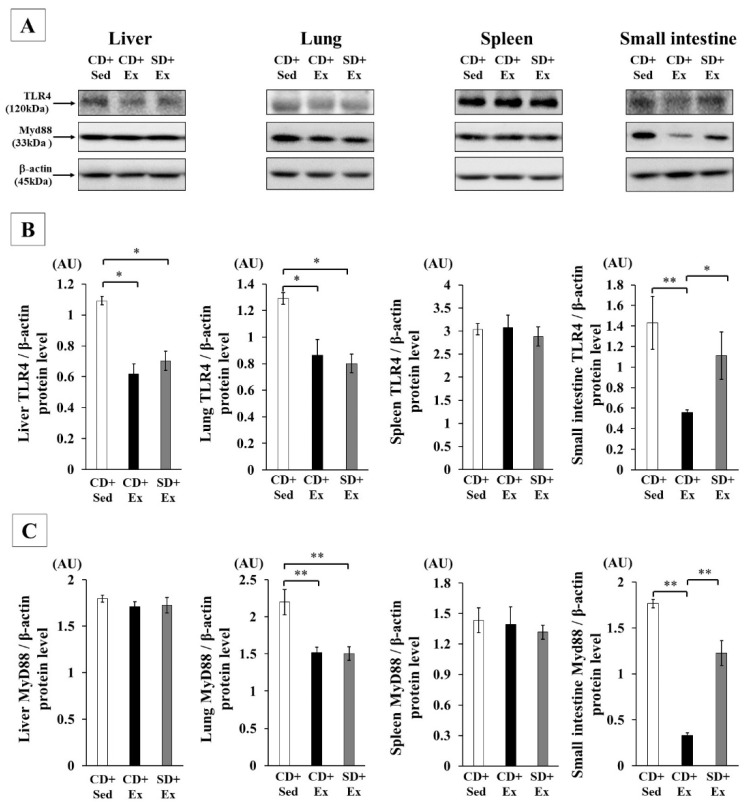
The effect of exhaustive exercise and *Sparassis crispa* intake on expression of TLR4 and MyD88 proteins in the liver, lung, spleen and small intestine of mice. Representative immunoblotting image and histograms of the levels of TLR4 and MyD88 are shown (**A**). β-actin protein expression was used as an internal control for normalizing the protein expression of TLR4 (**B**) and MyD88 (**C**) in the liver, lung, spleen, large intestine and small intestine. AU, arbitrary units. Values are means ± SEM. * *p* < 0.05, ** *p* < 0.01.

MyD88 protein expression levels in the lung and small intestine of CD + Ex group mice were significantly lower than those in the CD + Sed group, whereas the SD + Ex group exhibited a significant increase in MyD88 protein expression level in the small intestine compared with that in the CD + Ex group (Figure 3). No significant difference in MyD88 protein expression level was observed across the four groups in liver and spleen tissue (Figure 3).

## 4. Discussion

In this study, we revealed the preventive effects of continuous *S. crispa* intake on strenuous exercise-induced immunodepression. In this study, exhaustive exercise inhibited LPS-induced increases in plasma TNF-α levels. However, continuous *S. crispa* intake attenuated the exhaustive exercise-induced reduction of TNF-α production in response to LPS. Additionally, tissue TNF-α levels in the liver, spleen, lung and small intestine were significantly decreased by exhaustive exercise, whereas continuous *S. crispa* intake attenuated the reduction of LPS-induced TNF-α production in the small intestine after exhaustive exercise. Thus, the immunopotentiative effect of continuous *S. crispa* intake in the small intestine may contribute to the enhanced effect on LPS-induced TNF-α production after exhaustive exercise.

LPS binds to TLR4, which leads to activation of MyD88 and nuclear factor-κB (NF-κB) phosphorylation, and then induces the production of inflammatory cytokines, such as TNF-α [13]. In this study, as a result of confirming LPS-induced activation of the TLR4 signaling pathway when investigating the molecular mechanism underlying the effect of *S. crispa*, levels of TLR4 and MyD88 protein expression were decreased in the liver, lung and small intestine by exhaustive exercise. In the small intestine, however, the decreases in levels of TLR4 and MyD88 protein expression after exhaustive exercise were attenuated by continuous *S. crispa* intake. Thus, the retention of TLR4 and MyD88 protein levels in the small intestine may be involved in the preventive effect of continuous *S. crispa* intake on reduced LPS-induced TNF-α production after exhaustive exercise.

In this study, continuous *S. crispa* intake helped promote LPS-induced TNF-α production after exhaustive exercise. However, it is unclear what nutrients in *S. crispa* resulted in this effect. A previous study reported that *S. crispa* contains a high amount of β-glucan as compared with levels in other mushrooms [6]. In a cell culture study, β-glucans isolated from *S. crispa* were found to enhance LPS-induced inflammatory cytokine (TNF-α, IL-1β and IL-12) production in dendritic cells [14]. In agreement with this, we showed that continuous *S. crispa* intake attenuated the reduction of LPS-induced TNF-α production that normally occurs after exhaustive exercise. Thus, β-glucans present in *S. crispa* may lead to improvement of TNF-α production in response to LPS after exhaustive exercise.

We showed that exhaustive exercise inhibited LPS-induced TNF-α production in the liver, spleen, lung and small intestine. These findings agreed with the results of our previous study [3]. Moreover, in the present study, continuous *S. crispa* intake attenuated this reduction of LPS-induced TNF-α production, and resulted in retained levels of TLR4 protein expression after exhaustive exercise in the small intestine. Previous studies have reported that β-glucan administration increases NF-κB activation in small intestinal leukocytes and enterocytes in the small intestine [15]. Additionally, the increased number of lymphocytes in the small intestine by β-glucan administration may be related to increased systemic TNF-α production [16]. Thus, since leukocytes and enterocytes in the small intestine are involved, continuous *S. crispa* intake may enhance LPS-induced TNF-α production in the small intestine. However, in this study, we did not isolate these specific cells and analyze their function in exhaustive-exercised mice, so we cannot comment on the effect that *S. crispa* intake may have on leukocyte and enterocyte function in the small intestine. Further studies should examine the effect of *S. crispa* on these cells.

Intense exercise-induced immunodepression decreases resistance to pathogens, resulting in increased risk of infection and illness in athletes and exercise enthusiasts [2,17,18]. Nutritional interventions effectively improve immune cell dynamics in blood and promote healthy basal levels of cytokines and antibody production after intense exercise or training [1,19,20]. In this study, continuous *S. crispa* intake attenuated the reduction of the LPS-induced immune reaction after exhaustive exercise via upregulation of TLR4. TLR4 deficiency increases mortality and reduces bacteria clearance after an infection [21], so TLR4 function plays a critical role for the immune response during bacterial infection [13]. Thus, continuous *S. crispa* intake contributes to the host immune response when defending against a bacterial infection. These findings suggest that *S. crispa* may serve as an effective nutritional supplement for athletes and exercise enthusiasts under heavy physical training.

The continuous intake of *S. crispa* attenuated the reduction of LPS-induced TNF-α production and level of TLR4 protein expression after exhaustive exercise only in the small intestine. A previous study indicated that oral administration of dry mushroom powder increases the level of TLR4 mRNA expression in the small intestine [22]. Additionally, Volman et al. demonstrated that oral administration of β-glucan increases nuclear translocation of NF-κB in small intestinal leukocytes and enterocytes [15]. Moreover, Shen et al. suggested that an increase in the number of lymphocytes in the small intestine after oral administration of β-glucan may cause an increase in systemic TNF-α production [16]. Since leukocytes and enterocytes of the small intestine are involved, continuous *S. crispa* consumption may enhance the production of LPS induced-TNF-α only in the small intestine. However, in this study, we neither analyzed the activation of NF-κB nor isolated the leukocytes and enterocytes from the small intestine of exhaustively exercised mice. Because activation of NF-κB plays an important role in inflammatory cytokine production in response to LPS, future studies should examine the nuclear translocation of NF-κB and isolation of the abovementioned cells from the small intestine after exhaustive exercise.

## 5. Conclusions

These findings of this study revealed that continuous *S. crispa* intake prevents the reduction of LPS-induced TNF-α production that occurs after exhaustive exercise, and may exert this effect by ameliorating TLR4 and MyD88 protein levels in the small intestine.

## Figures and Tables

**Table 1:** Values are means ± SEM. CD + Sed: control diet + sedentary group, CD + Ex: control diet + exercise group, SD + Ex: *Sparassis crispa* diet + exercise group.

	CD + Sed (n = 8)	CD + Ex (n = 10)	SD + Ex (n = 10)
Body weight (g)	27.82 ± 0.66	28.10 ± 0.25	27.53 ± 0.36
Heart mass (mg)	106 ± 2	109 ± 3	110 ± 3
Gastrocnemius muscle mass (mg)	112 ± 4	115 ± 4	118 ± 5
Soleus muscle mass (mg)	6 ± 1	7 ± 1	6 ± 1
Plantaris muscle mass (mg)	5 ± 1	17 ± 1	14 ± 1
Tibialis anterior muscle mass (mg)	43 ± 1	44 ± 2	43 ± 1
Food intake (g / day)	3.19 ± 0.66	3.18 ± 0.28	3.18 ± 0.16
